# Quality and quantity of sample size is crucial in clinical studies to exclude association: antimicrobial exposure and the risk of delirium in critically ill patients

**DOI:** 10.1186/s13054-019-2381-1

**Published:** 2019-04-29

**Authors:** Rashid Nadeem, Zainab Ameer Obeida, Sahish Kamat, Ahmed Nazir Elsousi

**Affiliations:** Dubai Hospital, P O Box 7272, Dubai, United Arab Emirates

**Keywords:** Delirium, Cefepime, Antibiotics

To the Editor:

We read with great interest the article published in a recent issue of *Critical Care* by Grahl et al. [1]. The authors illustrated that there is no association between delirium and cefepime, penicillin, carbapenems, fluoroquinolones, or macrolides.

We would like to point out following concerns about this study.

We believe sample was not representative of general population and sample size was not powered enough to draw these conclusions. Study sample does not include all consecutive subjects from a time frame; a large portion of subjects 96 records (18%) were excluded because medication data was not available. Intention to include analysis might change results significantly. Authors did not mention if excluded patients’ profile was similar to included patients’ profile. Delirium was documented in 318 (76%), which is significantly more than expected from a typical ICU which suggests that sample may not be representative of general population. Sample predominantly comprises of Caucasian population (88%) so results may not be applicable to African American population.

This study found no association between delirium and cefipime. Although the analysis is performed for cefipime as individual drug, actual number of patients treated with cefipime was not provided; the key component to determine this association. Most likely no association was found as the number was inadequate. This is a common phenomenon; a negative study because of small sample size. Had they analyzed all patients on all cephalosporins including cefepime as one group, they may find association. Conversely they analyzed all other antibiotics (vancomycin, antifungal, antiviral and others) as one group and found them to be associated with delirium. Had they analyzed these antibiotics individually (like cefepime) these antibiotics will also not be associated with delirium as well (sampling error). We believe sample size for patients with cefipime may not be enough to exclude its association with delirium. Moreover median days of use of cefipime was 4.5 days in this study which suggest that physicians might have stopped the drug because of delirium as it is known that delirious effect from cefipime manifest after about 4 days of utilization. It will be interesting if data is available regarding how many patients were on cefipime and how many of them stopped cefepime because of delirium.

Sample also exclude patients with any neurologic disorder, yet 56% were comatose, and analysis showed no association of delirium with sedatives or opiates. Again it only suggests that sample is not large enough to answer these questions. We have to ask ourselves. Was study powered enough to exclude cefipime as associated factor for delirium?

We would also like to express clearly that previously published data which associate cefipime with delirium is also small, heterogeneous and insufficient and mainly comprise of case reports, case series and meta-analysis [2, 3]. To our knowledge, there is no randomized, prospective trial till date, therefore we believe this question remained unanswered until we have a well design study that is powered enough to answer this important clinical question. Clinicians are advised to exercise their best clinical judgement.
