# Supplementary material for: Quality and quantity of sample size is crucial in clinical studies to exclude association: antimicrobial exposure and the risk of delirium in critically ill patients
Source: Crit Care. 2019 Apr 29;23:147. doi: 10.1186/s13054-019-2381-1 (PMC6487514; doi:10.1186/s13054-019-2381-1)
Supplement: Related texts

# Reply: Authors’ response

**Authors:** Jessica J. Grahl, Joanna L. Stollings, Mayur B. Patel

We read with interest the comments by Nadeem et al. regarding the results of a nested cohort study from the BRAIN-ICU Study evaluating the association between antimicrobial class exposure in critically ill patients and the risk of delirium [4, 5]. After multiple covariate adjustment, only first- to third-generation cephalosporins were associated with delirium.

We agree that the sample size in this study [1] was small and an adequately powered study may provide different findings. However, we believe our cohort was truly reflective of the general ICU population. Delirium occurred in 308 (74%) of patients in this cohort, which was appropriate for the study time frame (March 2007–May 2010). Delirium recognition and prevention strategies have improved since guideline publications and the ICU Liberation ABCDEF Bundle implementation [6, 7].

Cefepime was utilized in 64 (15%) of the patients that received antimicrobial therapy. This information is listed in supplementary data (Table S1) [4]. We conducted an unpublished statistical analysis looking at beta-lactams as a class and found a statistically significant difference, which is why sub-class analysis was conducted. In order to prevent statistical model overfitting, it was necessary to include a class of “other antimicrobials”, as opposed to individualized therapy and covariate adjustments for these multitude of antibiotics without a strong past scientific association with delirium. Although our approach is imperfect, when studying cefepime, all other past studies lack adjustment for competing classes of antibiotics.

It is unlikely that providers would have discontinued therapy due to the incidence of delirium alone. At the time this study was conducted, there was no literature identifying an association between cefepime and delirium based on CAM-ICU monitoring. Rather, we credit the median duration of cefepime of only 4.5 days to twenty-first century antimicrobial stewardship.

We also excluded patients with severe neurological disease, which is common practice in many studies evaluating an association between pharmacotherapy and delirium [5, 8] We agree the findings may not be extrapolatable to this patient population. We did not identify an association with use of analgesics and sedatives in the ICU in our proportional odds model. This is most likely because the covariates were binary outcomes measured on ICU admission.

The authors agree with Nadeem et al. that the previously published data associating cefepime with delirium are small, heterogenous, and mostly case reports and series. The current study is the first to date that sought to find an association between antimicrobial therapy and delirium using the CAM-ICU assessment. We agree that future adequately powered studies are warranted. However, we remain leery of reflexively shifting active antimicrobial strategies simply because of the occurrence of delirium.
